# Transposon-Based Reporter Marking Provides Functional Evidence for Intercellular Bridges in the Male Germline of Rabbits

**DOI:** 10.1371/journal.pone.0154489

**Published:** 2016-05-05

**Authors:** Orsolya I. Hoffmann, Andrea Kerekes, Nandor Lipták, Laszlo Hiripi, Szilard Bodo, Gabor Szaloki, Sabine Klein, Zoltan Ivics, Wilfried A. Kues, Zsuzsanna Bosze

**Affiliations:** 1 NARIC-Agricultural Biotechnology Institute, Gödöllő, Hungary; 2 Faculty of Medicine, University of Debrecen, Department of Biophysics and Cell Biology, Debrecen, Hungary; 3 Department of Biotechnology, Friedrich-Loeffler-Institut, Institut für Nutztiergenetik, Mariensee, Neustadt, Germany; 4 Paul-Ehrlich-Institut (PEI), Langen, Germany; University of Birmingham, UNITED KINGDOM

## Abstract

The *Sleeping Beauty* transposon system was established as a robust and efficient method for germline transgenesis in different mammalian species. The generation of transgenic mice, rats, rabbits and swine carrying an identical Venus reporter construct delivered by transposon-mediated gene transfer enables comparative studies of gene expression in these lines of mammalian models. Whereas comparable expression patterns of the Venus reporter were found in somatic tissues, preliminary studies suggested that a striking difference in reporter expression may exist in mature spermatozoa of these species. Here we clearly show the differential expression of Venus reporter protein during spermatogenesis of the two compared species, the laboratory rabbit and mice. We provide evidence for the functionality of intercellular bridges in the male germline and genotype-independent transgenic phenotype of rabbit spermatids. Our data suggest that the reporter rabbit line may be a suitable tool to identify molecular mechanisms in testicular development, and may contribute to develop better animal models for male infertility in men.

## Introduction

The laboratory rabbit (*Oryctolagus cuniculus*) is the third most widely used laboratory mammal, after mice and rats [1]. Traditionally, the rabbit has been the most commonly used species of fertilization research, resulting in the first descriptions of sperm and oocyte transport, prenatal mortality, oocyte maturation and fertilization, genetics of gametes and mature spermatozoa morphology[2]. The rabbit model is still indispensable to examine aspects of embryonal development, diabetic pregnancy, male infertility, and specific human diseases [2,3]. Women, mouse and rabbits have haemochorial placenta. Rabbits possess haemodichorial placenta which is anatomically closer to the human haemomonochorial placenta than that of the mouse (haemotrichorial)[4]. In surgical experiments, rabbit is better animal model than mouse due to its relative large size.

A clear bottleneck for a broader application of the rabbit model was the limited possibilities for genetic modifications in this species, relative to the mouse model[2,3]. The recent introduction of advanced methods for genetic engineering by hyperactive transposases and designer nucleases [5,6] changed this scenario completely, allowing the rapid and efficient generation of genetically modified rabbits [7,8]. Moreover, the current toolbox for genetic engineering allows comparative studies between different model species, such as mouse and rabbit to elucidate common and unique features of transgenesis.

Recently, the hyperactive variant of a DNA class II transposon, *Sleeping Beauty* (SB100X) has been successfully applied for transgenesis for the first time in the laboratory rabbit [8,9]. In this binary system, the SB100X transposase is supplied as mRNA or as helper plasmid (pCMV-SB100X) and the transposon plasmid that carries the transgene, for example an SB inverted terminal repeat (ITR) flanked Venus fluorophore coding sequence driven by the ubiquitous CAGGS (CAG) promoter [5]. In parallel, the same CAG-Venus transgene was transposed in mouse, rat, and pig species [8–12] providing us with the unique opportunity to perform comparative studies between these model species.

The Venus reporter protein was expressed in all examined organs and tissues of all four SB-transgenic species. The published data underline the advantage of the SB transposase catalyzed gene delivery compared to the classical microinjection method [13]. The SB transposon, contrary to viral-based methods, has a close-to-random insertion profile in mammalian cells [14,15]. Moreover, the 15% founder rate with 100% germline transmission in laboratory rabbit made SB transgenesis the most efficient method for additive transgenesis in this species [9]. SB transgenesis in rabbit therefore could be instrumental in creating novel disease models.

Our previous observation revealed differential expression pattern of Venus in the testes of SB transgenic mice and rabbits; Venus expression was found to be restricted to Leydig cells in mice (four founders and eight offspring), contrary to rabbit where expression was not confined to Leydig cells (one founder, six offspring). In parallel, it was published that the mature spermatozoa of founder and F1 CAG-Venus SB transgenic boars were loaded with Venus reporter protein independent from the genotype [10]. Those observations led us to examine the species-specificity of Venus protein expression in prospermatogonial stages and in mature spermatozoa in SB-CAG-Venus transgenic rabbits and mice. The potential influence of an ectopic protein expression on sperm quality and the SB transgenic buck’s litter size was also evaluated. Transgenic rabbits expressing fluorescent reporters (EGFP) under control of the CAG promoter were already created and characterized earlier e.g. for organ transplantation, however transgene expression in the ejaculated spermatozoa have not been analysed before [16–18]. Here we clearly show the differential expression of Venus reporter protein during spermatogenesis of the two compared species, the laboratory rabbit and mice.

## Materials and Methods

### Ethics statement

All experiments were approved by the Animal Care and Ethics Committee of the NARIC-Agricultural Biotechnology Institute and the Pest County’s governmental office (permission numbers: PEI/001/329-4/2013; PEI/01/857-3/2015). The experiments were complied with the Hungarian Code of Practice for the Care and Use of Animals for Scientific Purposes, including conditions for animal welfare and handling prior to slaughter.

### Flow cytometry

**Fig 1:**
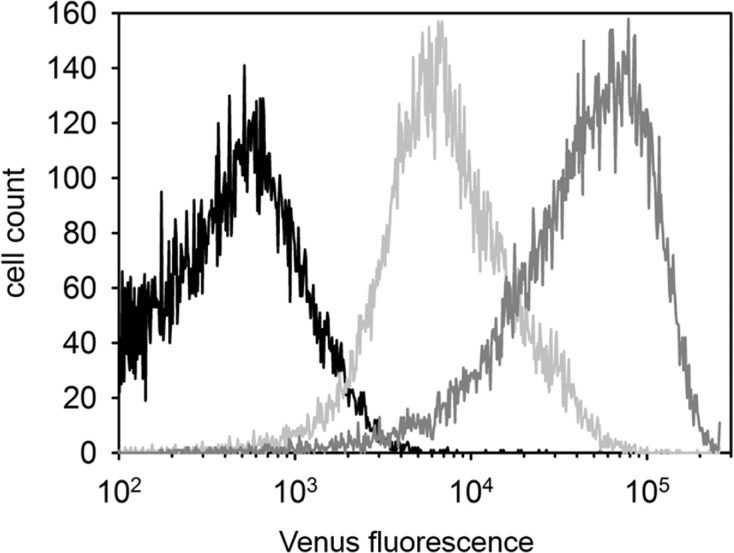
Differential Venus expression in the SB-CAG-VENUS rabbit spermatozoa (FACS). Semen derived from a wild type (black), a hemizygous (# 4004, light gray) and a homozygous (# 4007, gray) buck.

Semen samples were fixed in 0.5% buffered formaldehyde and stained with Hoechst 33342. Venus fluorescence measurements were carried out on a BectonDickinson FACSAria(TM) III Cell Sorter (Becton Dickinson, Mountain View, CA, USA). The Venus was excited by the 488 nm line of an Ar-ion laser and the emitted light was detected using a 502 nm dichroic mirror and a 530/30 nm band-pass filter. The Hoechst 33342 was excited by a 375 nm solid state UV laser and it was detected using a 450/40 nm, band-pass filter. Flow cytometric data were analysed by using BD FACSDiva Software (BectonDickinson, Mountain View, CA, USA) and FCS Express 4 Research Edition (DeNovo Software, Glendale, CA, USA). *Fig 1* was made using SigmaPlot 12.5 (Systat Software, Inc., CA, USA).

### Histology

The rabbit testis and mouse epididimys tissue samples were fixed with 4% buffered formaldehyde, and cryoprotected in 30% sucrose solution. Afterwards, the samples were embedded in cryostat-embedding compound (Tissue-Tek, Torrance, CA) and cut into 10-μm thick sections on a cryostat (Microm, Heidelberg, Germany). The samples were nuclear stained with TO-PRO-3 Iodide (Thermo Fisher Scientific, T3605) and covered with FluoroSave Reagent (Merck Millipore).

### Immunohistology to detect intercellular bridges in rabbit testes

The immunohistology were performed on 10-μm thick sections of rabbit testis. Briefly, after a basic heat-induced epitope retrieval protocol, the sections were blocked with 5% BSA and incubated overnight with Tex-14 polyclonal goat primary antibody (Santa Cruz Biotechnology Inc., sc169574). The primary antibody was removed by recurring washing with TBST. The fluorescent donkey anti-goat secondary antibody (Thermo Fisher Scientific, A-21082) was incubated for one hour and the remainder was washed away. The nuclei were visualised with a 30 minute staining (TO-PRO-3 Iodide, Thermo Fisher Scientific, T3605) and the slides were covered with a mounting media (FluorSave Reagent, Merck Millipore).

### Fluorescence microscopy

The images were obtained with a Leica TCS SP8 confocal microscope equipped with PMT detector. The detection range of the Venus channel was 510–550 nm. The nuclear staining was recorded at 650–726 nm. In case of the TEX-14 immunohistochemistry the secondary antibody was detected at 640–682 nm.

### RT-PCR from rabbit semen

Semen was collected from both TG and non-TG bucks using artificial vagina.

Somatic cells were removed from ejaculated sperm by percoll gradient (90%) centrifugation for one hour as described in [10]. RNA was purified from the separated spermatozoa fraction by RNAzol RT reagent (MRC) according to the manufacturer’s instructions. cDNA were reverse transcribed with Applied Biosystems High-capacity cDNA Reverse Transcription Kit (Life Technologies) from 200 ng RNA. The RT-PCR reactions were set up with MyTaq Red Mix (Bioline) reagent according to the manufacturer’s instructions. The following primer pairs were used to RT-PCR:

Venus specific primer: Forward: 5’ GGTCCCTCTTCTCGTTAGGG 3’

Reverse: 5’ TACAAGACCAGAGCCGAGGT 3’;

Neonatal rabbit Fc receptor specific primer [19];

Ribosomal 28S subunit specific primer, Forward: 5' GTTGTTGCCATGGTAATCCTGCTCAGT 3', Reverse: 5' TCTGACTTAGAGGCGTTCAGTCATAAT 3'

### CASA measurements and spermatozoa viability

Motility parameters of spermatozoa were exactly measured with computer-assisted sperm analyser (CASA, SpermVision Minitube). The results of CASA contain the percentage of the total mobile cells (MOT), the progressive motility (PMOT), the straight-line velocity (VSL), curvilinear velocity (VCL) and the curvilinear trajectory (LIN). The viability, acrosome and tail membrane integrity staining of the freshly ejaculated spermatozoa was evaluated with Kovacs-Foote staining [20,21].

### Western blotting

Samples were diluted with physiological salt to 2x10^9^ spematozoa/ml. The protein fraction were extracted from the percoll gradient purified spermatozoa with extraction puffer (20mM HEPES pH 7.5; 420 mM NaCl; 1 mM EDTA; 25 mM DTT; 25% glicerin). The samples were separated on denaturing 12% SDS–polyacrylamide gel and were blotted to PVDF membrane (Hybond-P, Amersham). After the overnight blocking with 5% glycerine defatted dry milk, the membrane were incubated for 4 hours at 4°C with an anti-EGFP polyclonal antibody (1:2000, Thermo Fisher Scientific), then incubated for 2 hours at 4°C with a horseradish peroxidase conjugated anti-rabbit-IgG secondary antibody (1:10000, Sigma A5906). The blots were developed using the ECL-Advanced chemiluminescence detection system (Amersham) on Hyperfilm ECL autoradiography film (Amersham).

## Results

### Venus flurophore expression in spermatozoa

Out of the seven SB transposon transgenic founder rabbits (four males, three females), the # SB3JT was used to establish and maintain a transgenic line. The hemizygote rabbits of this SB-CAG-Venus transgenic line carry one monomeric transgene at chr8 [8].

Flow cytometric measurements detected prominent Venus-fluorescence in the spermatozoa of # SB3JT males (*Fig 1).*

Notably, the FACS analysis showed an intensity difference between the hemizygote (#4004) and homozygote (# 4007) spermatozoa (*Fig 1*). The genotypes of the transgenic bucks were confirmed both by Q-PCR (data not shown) and by Mendelian segregation of the Venus reporter when mated with wild type and hemizygote transgenic does (*Table 1*).

**Table 1:** Comparative data on litter size and transgene inheritance SB-CAG-Venus hemi- and homozygote bucks and in two independent transgenic lines, in which the transgene product is not expressed in the spermatozoa.

*Buck genotype*	*Transgenic line*	*Does genotype*	*Litter size*	*Number of transgenic offspring*
#4004 hemizygote	SB-CAG-Venus (SB3JT)	wild type	7	4
#4004 hemizygote	SB-CAG-Venus (SB3JT)	#4011 hemizygote	8	5
#4004 hemizygote	SB-CAG-Venus (SB3JT)	#4014 hemizygote	5	2
#4007 homozygote	SB-CAG-Venus (SB3JT)	wild type	5	5
#4007 homozygote	SB-CAG-Venus (SB3JT)	wild type	8	8
#4007 homozygote	SB-CAG-Venus (SB3JT)	#4010 hemizygote	5	5
#4007 homozygote	SB-CAG-Venus (SB3JT)	#4013 hemizygote	5	5
#3005 hemizygote	WAP-hTNAP	wild type	8	3
#3005 hemizygote	WAP-hTNAP	wild type	10	4
#3005 hemizygote	WAP-hTNAP	#3003 hemizygote	7	5
#3009 homozygote	WAP-hTNAP	wild type	5	5
#3009 homozygote	WAP-hTNAP	wild type	3	3
#5115 hemizygote	β-MHC-G52R-KCNE1	wild type	4	1
#5115 hemizygote	β-MHC-G52R-KCNE1	wild type	12	6

**Fig 2:**
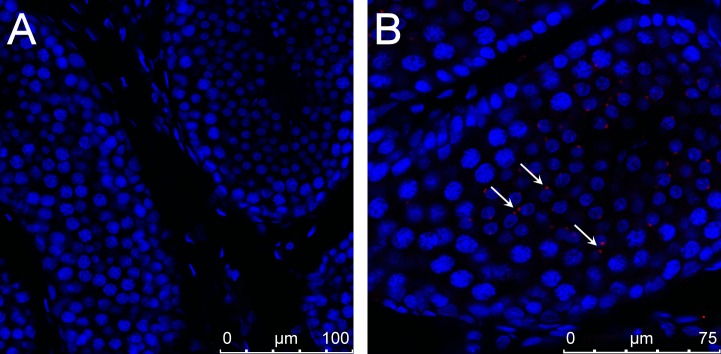
Detection of TEX-14 the critical component of mammalian intercellular bridges in the SB-CAG-Venus rabbit testis. The image of TEX-14 staining in rabbit testis (red). The section was counterstained with TO-PRO-3 Iodide (blue). A: control, without primary antibody, B: Immunostaining with primary (TEX-14 polyclonal goat) and secondary (donkey anti-goat, Alexa Fluor 633) antibody. The presence of TEX-14 protein in the intercellular bridges is indicated by arrows.

It should be emphasized that, although the Venus fluorescence of the hemizygote sperms is less intensive compared to the homozygote’s, all hemizygote sperm cells were found uniformly Venus-positive with the FACS analysis (*Fig 1*). Because only 50% of all sperm cells of a hemizygous male are expected to carry the Venus transgene, this observation points to the functional role of intercellular bridges, which exist between spermatocytes and spermatids and which could explain the even distribution of the Venus fluorophore protein between transgenic and non-transgenic sperm cells. Since the first electron microscopic observations [22], no novel data on the structural components of intercellular bridges in the rabbit female and male germline were described. Here we detected the TEX-14 protein, required for stable intercellular bridge formation [23] in the # SB3JT transgenic rabbit’ s testis with immunohistology (*Fig 2*).

### Developmental stage-dependent expression of Venus fluorophore in rabbit testis

**Fig 3:**
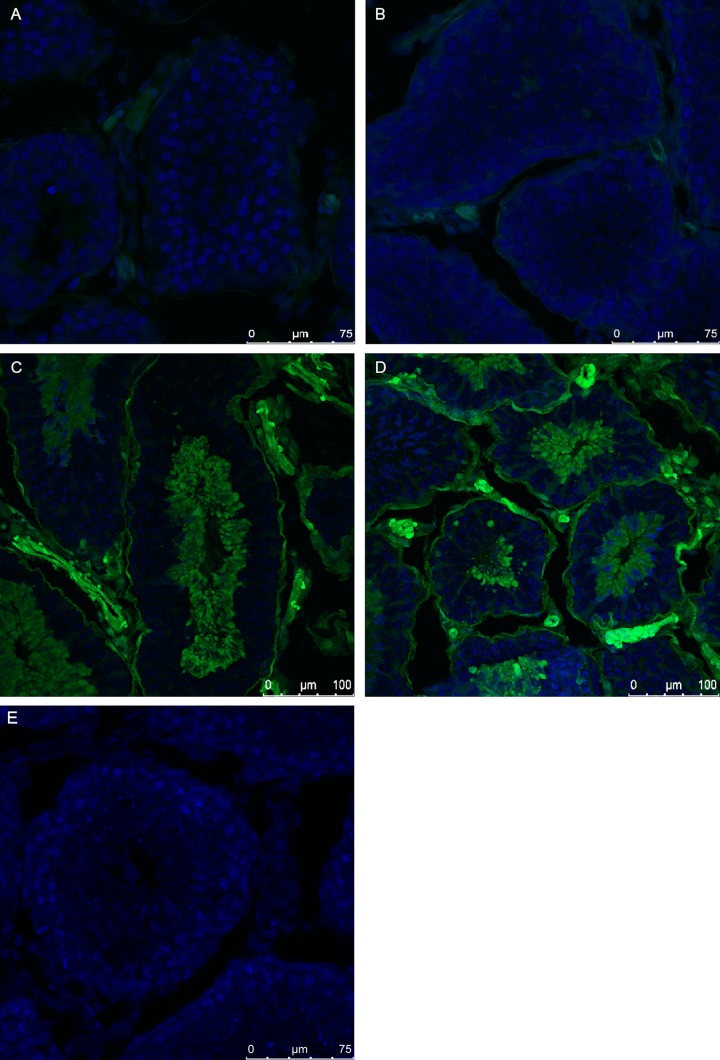
Comparison of Venus expression in the developing testis of # SB3JT bucks. Venus expression shows an increasing pattern in the 42 days hemizygous (A), 60 days hemizygous (B) 120 days old hemizygous (C) and homozygous testis (D). The control testis (E) does not show detectable Venus expression.

Taking advantage of the traceability of Venus expression, developmental stage-specific expression of this ectopic protein was examined. The postnatal testicular development of rabbit is well described and although it reveals some rabbit breed specific differences, overall its timing resembles that of human [24–26]. Therefore, we compared the testes of young bucks of the # SB3JT line at the pre-spermatogenesis stage (42dpp), at 60 dpp, the beginning of spermatogenesis and in adult buck’s testis (120 dpp). At 42 dpp the differentiating germ cells do not express the Venus protein at detectable levels, contrary to the somatic cells, which do show transgene expression *(Fig 3A*).

At 60 dpp the differentiation to spermatogonia has started and accompanied by faint Venus expression (Fig 3B). In the adult testis Venus-expressing spermatocytes, and round and elongated spermatids were identified both in hemi- (Fig 3C) and homozygote bucks (Fig 3D). Moreover, the interstitial tissue, Leydig cells and smooth muscle cells were also Venus-positive (Fig 3C and 3D). Based on the obtained information it could be concluded, that Venus expression in the SB-CAG rabbit male germ line begin in the spermatogonial cells and became uniformly strong in spermatocytes and spermatids. The non-transgenic littermate buck’s testis did not show Venus expression (Fig 3E).

### Detection of Venus mRNA in mature rabbit spermatozoa

**Fig 4:**
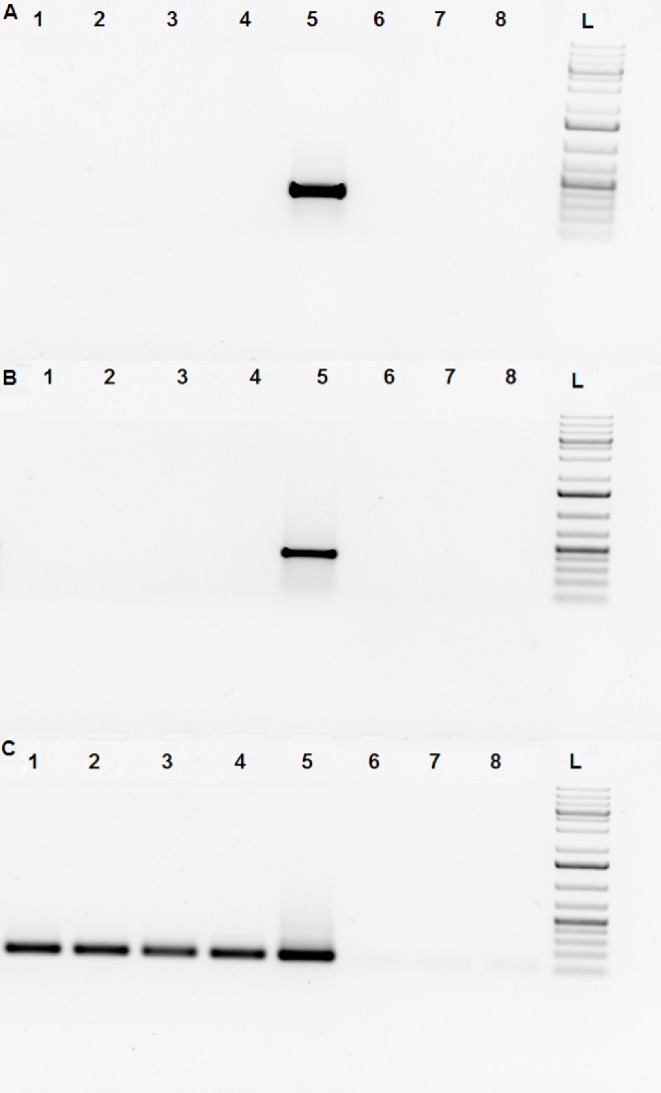
Absence of Venus transcript in SB-CAG-VENUS spermatozoa. The following primer pairs were used in RT-PCR experiments: Fig 4A: YFP Venus specific primer:Forward: 5’ GGTCCCTCTTCTCGTTAGGG 3’ Reverse: 5’ TACAAGACCAGAGCCGAGGT 3’ Fig 4B: neonatal rabbit Fc receptor specific primer [19]; Fig 4C: ribosomal 28S subunit specific primer.Forward:5'GTTGTTGCCATGGTAATCCTGCTCAGT 3' Reverse: 5' TCTGACTTAGAGGCGTTCAGTCATAAT 3' RT-PCR of the buck’s sperm samples after percoll purification: **Line 1**: control wild type spermatozoa cDNA, **Line 2**: #4020 homozygote SB-CAG-Venus spermatozoa cDNA, **Line 3, 4**: #4017, #4012 hemizygote SB-CAG-Venus spermatozoa cDNA (percoll purified), **Line 5**: reference SB-CAG-Venus fibroblast, **Line 6**: no template RT-PCR, **Line 7**: no enzyme RT-PCR, **Line 8**: water control RT-PCR, L: DNA Ladder (GeneRuler 1Kb Plus). Venus transcripts were only detected in SB-CAG-Venus fibroblast samples.

To determine if Venus fluorescence of mature sperm cells reflects active transcription, the ejaculated semen samples of # SB3JT bucks were centrifuged over a 90% percoll gradient to remove epithelial and leukocyte cells. Total RNA was isolated from the purified sperm cell fraction (*Fig 4A).*

The control RT-PCR reactions, one specific for the rabbit neonatal Fc receptor mRNA [19], which is expressed in epithelial cells, fibroblasts and leukocytes *(Fig 4B)* and another for the 28 S ribosomal RNA *(Fig 4C)* underlined the successful isolation of sperm-specific RNA. Venus-specific RT-PCR reproducibly failed to amplify the mRNA from sperm samples, thereby suggesting that neither newly transcribed nor remnant transgenic specific mRNA was present *(Fig 4A).* Western blot analysis confirmed the presence of the Venus protein in percoll purified spermatozoa of SB-CAG-Venus transgenic bucks, and mirrored the quantitative difference between the homo- and hemizygote sperm cells (*S1 Fig*). The obtained RT-PCR data point to the functional role of intercellular bridges in sharing the transgenic protein product between syncytias.

### Compartmentalized distribution of Venus fluorophore in the mature rabbit spermatozoa

**Fig 5:**
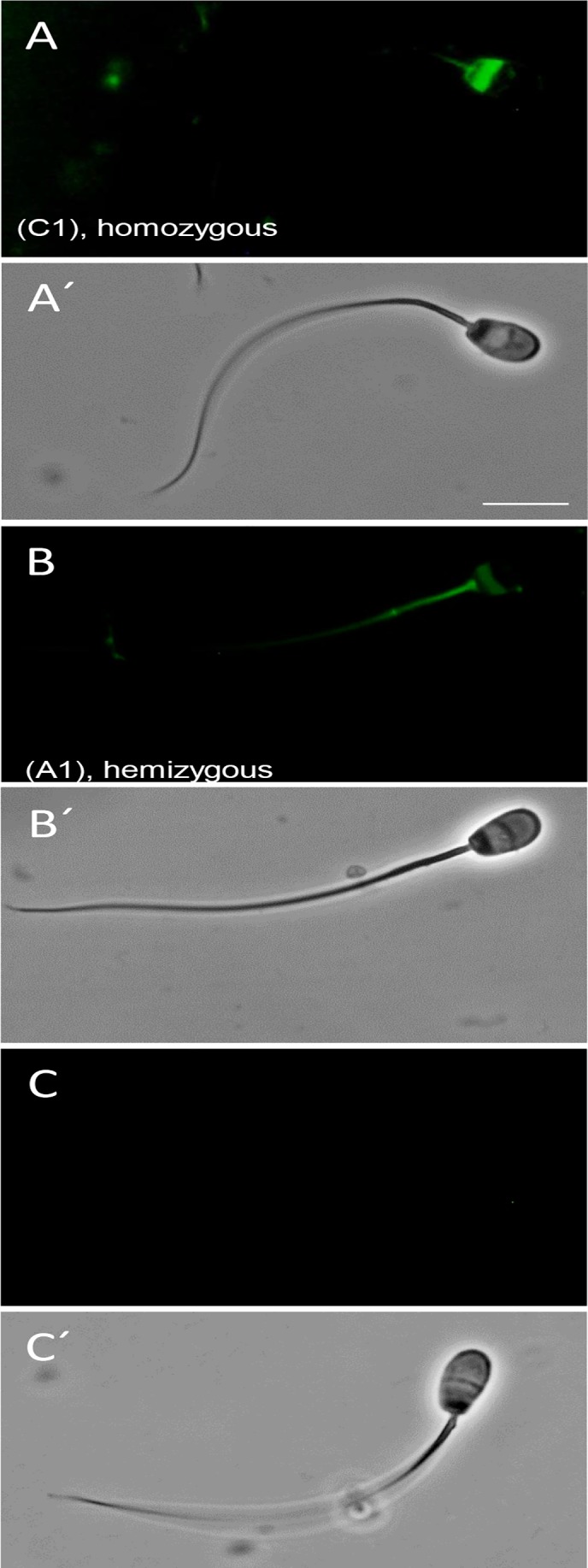
Venus expression in rabbit spermatozoa Representative, normalized Venus fluorophore expression in spermatozoa of # 4007 homozygous (A) and # 4004 hemizygous bucks (B) no expression in wild type littermate (C), viewed under specific excitation and dim bright light. A’, B’ and C’ shows the corresponding sperm under bright light.

The Venus expression detected with flow cytometric measurements (*Fig 1*) was apparent in # SB3JT spermatozoa under fluorescent microscope, although the fluorescence showed a pronounced accumulation to the postacrosomal sheath (PAS), just below the equatorial rim, the midpiece and the tail (*Fig 5A and 5B*).

### The influence of transgenic modification on rabbit semen quality

To evaluate if the expression of an ectopic reporter protein in rabbit sperm cells or the transgenic status *per se* has an influence on sperm motility, we compared semen samples of hemizygote and homozygote # SB3JT bucks to semen samples of hemizygote bucks of two independent transgenic rabbit lines, in which transgene protein expression was restricted to the mammary gland (WAP-hTNAP transgene) [27] and the heart (β-MHC-G52R-KCNE1 transgene) [27,28]. *Table 2* shows that the essential sperm parameters for motility as evaluated by Computer Assisted Semen Analysis (CASA) and the viability were not different in semen of the three transgenic lines compared to the wild type control, except that the sperm cells of the two non-Venus expressing transgenic lines showed a slightly reduced obvious forward motility compared to control.

**Table 2:** Comparative analysis of semen parameters from transgenic and control bucks (CASA) Data represent the mean ± S.D., #4017, #4020 are the SB-CAG-Venus transgenic bucks; #5117, #3005 are transgenic bucks [27,28]. Asterisks denote significance differences compared to control bucks (one way anova, Scheffe post hoc, p < 0.05), n = 4 replicates. The percentage of live spermatozoas with intact acrosomes were determined with the Kovacs-Foote staining [20,21].

	Animal ID				
	Control	#4017	#4020	#5117	#3005
	wildtype	hemizygote	homozygote	hemizygote	Hemizygote
Expressed transgene	None	SB-CAG-Venus	SB-CAG-Venus	G52R-KCNE1	WAP-hTNAP
Total motility (%)	82.8 ± 8.6	75.6 ± 10.2	79.1 ± 9.38	86.8 ± 6.7	82.8 ± 8.6
Progressive motility (%)	68.7 ± 12.5	61.7 ± 16.08	64.3 ± 14.8	77.9 ± 8	70.1 ± 11.93
Straight-line velocity (μm/s)	51.1 ± 4.95	54.5 ± 12.3	54.1 ± 7.69	44.2 ± 2.1	42.8 ± 4.86
Curvilinear velocity (μm/s)	123.6 ± 3.31	132.3 ± 23.98	132.2 ± 11.32	135.3 ± 2.92	130.7 ± 4.45
Linearity	0.41 ± 0.0082	0.41 ± 0.0287	0.40 ± 0.0252	0.32 ± 0.0154	0.32 ± .0583
Live spermatozoa with intact acrosome (%)	81.52	79.52	70.95	60.19	63.14

Litter sizes from the hemi- and homozygote # SB3JT bucks, were similar to the ones found in the other two independent transgenic lines [27,28], suggesting that the presence of the Venus protein does not affect sperm vitality (*Table 1*).

### Species-specific expression of Venus fluorophore in SB transgenic mice

**Fig 6:**
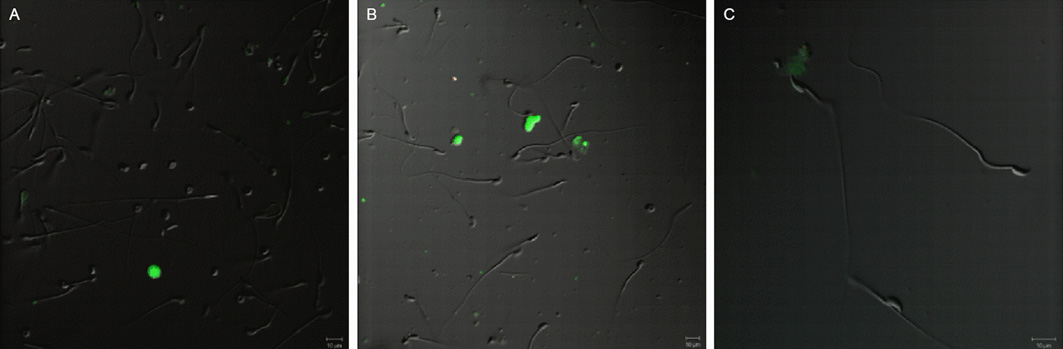
The lack of Venus fluorescence in spermatozoa of epididymis from three transgenic homozygote mouse strain. A:#VL1, B:#VL3, C:#VL4

Transgenic mice generated with the SB system containing a single copy of the SB-CAG-Venus transposon have been recently described [29]. Spermatozoa obtained from the epididymis of homozygous males of three different transgenic lines (VL1, VL3 and VL4) were Venus-negative (*Fig 6*) contrary to the transgenic pig’s [10] and rabbit’s (*Figs 1 and 5*), although those transgenic animals were created with the exact same transgene and technique.

**Fig 7:**
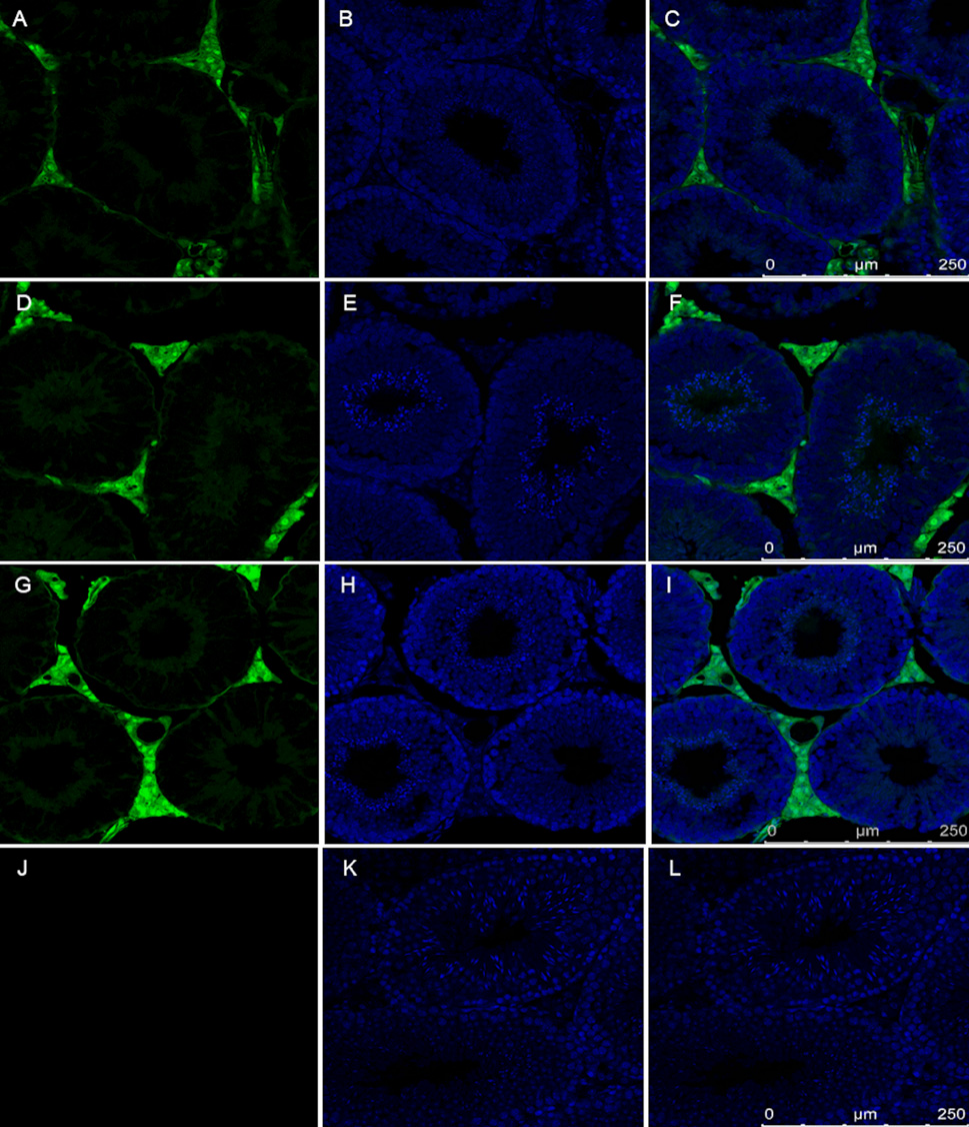
The expression of Venus protein of testis from three SB transgenic homozygote mouse lines The somatic cells expressed Venus protein, but spermatocytes, spermatids and spermatozoa did not show Venus specific fluorescence. Testis sections of wild type mice remained Venus negative. A,B,C:#VL1;D,E,F:#VL3;G,H,I: #VL4; J,K,L:wildtype

The mouse testis tissue sections revealed that the interstitial tissue, the Leydig cells and smooth muscle cells were strongly Venus positive, like in the adult transgenic rabbit. However, contrary to the rabbit (*Fig 3C and 3D*) the round and elongated spermatids and spermatozoa showed a very faint Venus fluorescence (*Fig 7*).

**Fig 8:**
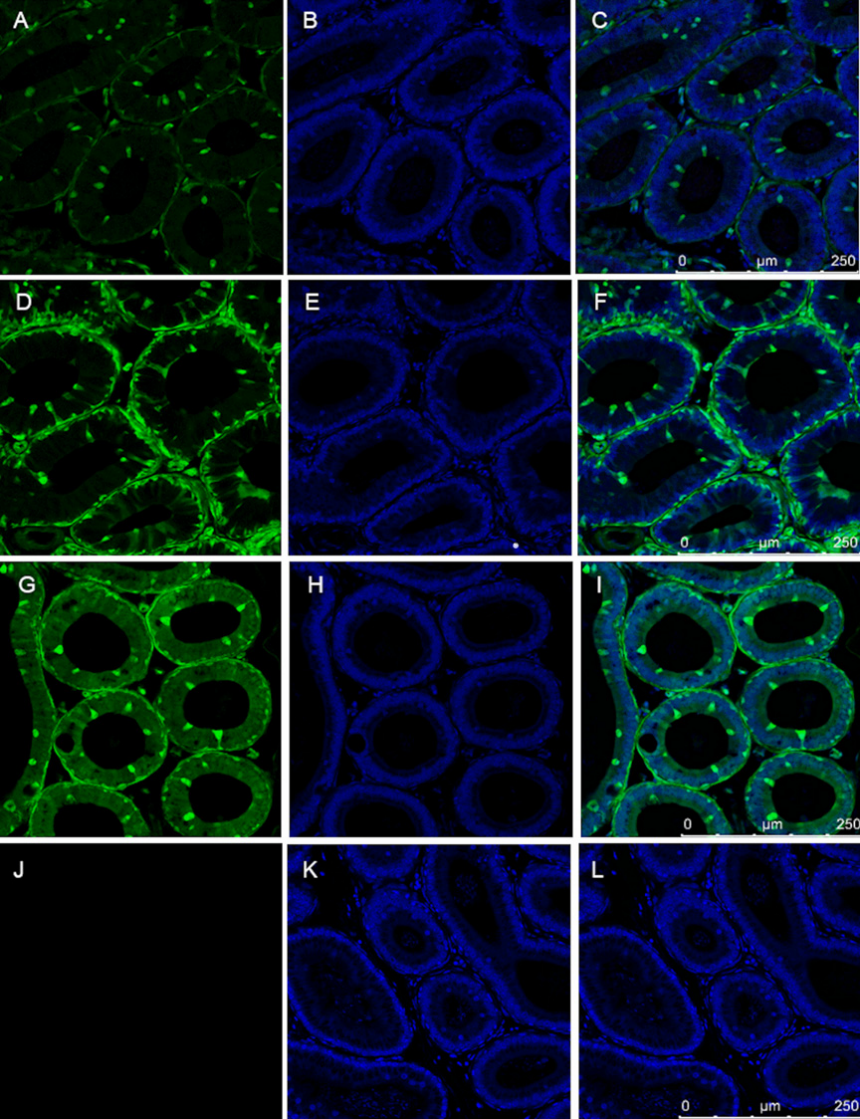
The expression of Venus protein in epididymis from three SB transgenic homozygote mouse strains. The somatic cells showed Venus specific fluorescence, but spermatozoa did not express Venus protein. Note the differential expression between the characteristic cell types, e.g. narrow/clear cells compared to principal and basal cells. Epididymis sections of wild type mice did not show Venus specific fluorescence.A,B,C:#VL1; D,E,F:#VL3; G,H,I:#VL4; J,K,L: wildtype

The epididymis is the place where spermatozoa undergo final maturation. The epididymal epithelium of SB-CAG-Venus males, which is composed of at least three cell types: narrow/clear, principal and basal cells revealed a complex Venus expression pattern (*Fig 8*).

Based on their characteristic morphological features, the narrow/clear cells and the interstitial cells highly express the Venus fluorophore in all three independent transgenic mouse lines.

## Discussion

Transposon-mediated genetic modification is an efficient way of additive transgenesis and enabled to create transgenic mouse, rat, rabbit and swine lines with the same SB-CAG-Venus transgene [8–12]. In general, similar expression patterns of the SB CAG-Venus reporter were found in somatic tissues of transgenic mice, rats, rabbit and swine, highlighting that the SB-mediated transgenesis robustly worked in a range of mammalian models. Importantly, the vast majority of these transgenic animals showed CAG promoter-dependent expression profiles of the reporter, whereas cases of variegated expression or position effects were rarely observed [29].

Previously, Venus fluorophore expression was analysed in detail in SB–transgenic boars and genotype independent expression was detected in the mature spermatozoa [10]. Surprisingly, preliminary observations indicated that SB-transgenic mouse spermatozoa did not express Venus. Those independent observations suggested that the terminally differentiated spermatozoa may represent an exception from the comparable expression pattern of the Venus reporter seen in somatic tissues of the SB transgenic species. Those observations lead us to examine Venus expression in spermatozoa and testis of SB-transgenic rabbits and mice, analysed by identical methods.

Here we show, that SB transgenesis with the CAG-Venus transgene resulted in fluorophore-expressing spermatocytes, spermatids and mature spermatozoa in the laboratory rabbit. The intensity of expression correlated with the transgene copy number, lining up with the observations made in the SB-transgenic pig lines [10]. In stark contrast, mouse spermatozoa obtained from the epididymis of three independent transgenic lines were negative for Venus expression. The difference in the overall intensity of Venus expression in the testis somatic cells between the three different mouse transgenic lines could be the consequence of the unique transgene integration sites, and the very faint Venus expression during sperm maturation could be due to the CAG promoter, which is directed by the trans-acting factors of each species and reflects species-specific aspects of spermatogenesis.

The CAG-EGFP transgenic rabbits created earlier with pronuclear microinjection by others were not analysed for EGFP fluorescence in the spermatozoa [16–18], whereas the semen samples of a cloned CAG-GFP transgenic boar were found negative for GFP fluorescence [18].

In our earlier experiments, lentiviral transgenesis in rabbit created with the CAG-GFP transgene resulted highly mosaic founders, which did not enable to establish transgenic lines, but in the testis of a founder buck a small fraction of spermatids expressed GFP [30].

Importantly, the CAG promoter was found to direct ubiquitous expression of GFP in all other organs of transgenic rats, rabbits and pigs [18].

Our observation that haploid spermatids of a hemizygote transgenic buck containing a single copy of the Venus transgene (that are therefore genetically distinct) all expressed the Venus protein suggests that either the transgenic mRNA or protein moved between the syncytial intercellular bridges. Genotype-independent transgenic phenotype of spermatids was first reported in a sperm specific protamine 1 promoter- human growth hormone transgenic hemizygote mouse model, where even distribution of transgene mRNA and protein were detected in testis sections [31]. Recently, genotype-independent transmission of Venus protein by spermatozoa was reported by [10] in SB- transgenic hemizygote transgenic boars, however to our knowledge beyond those two examples none were published in mammals.

Formation of intercellular bridges in the rabbit male germline were published [22,32], however evidence for the functionality of intercellular bridges in this species has not been reported to date. The testis-expressed gene 14 (TEX-14) is a marker for sperm cell intercellular bridges. TEX-14-/- mutant mice showed drastically lower numbers of spermatocytes, postmeiotic spermatids and spermatozoa [33]. Here we showed the presence of TEX-14 protein in the testes intercellular bridges of transgenic rabbits, and provide the first evidence of their function. We showed that hemi- and homozygote SB-transgenic spermatozoa carrying the ectopic Venus protein fulfilled the standard semen quality requirements. Indeed, these semen samples were used for artificial insemination through four generations, and neither reduced fertility nor smaller litter sizes were experienced.

Human male infertility is a regrettably common and complex problem. Male infertility may be caused by genetic, epigenetic, environmental, and nutritional factors, but the background of the male infertility often remains unknown. The important periods of pre-spermatogenesis and timing of testicular development of the laboratory rabbit are closer to human than to rodents. Thus, our results indicate that the SB-transgenic rabbit could be used as a model for detailed analysis of spermatogenesis. Alternatively, it might be interesting to create novel SB-transgenic rabbit lines, in which the fluorophore protein is combined with expression of a mutant human gene causing sterility.

## Supporting Information

S1 Fig Detection of Venus protein in SB-CAG-Venus buck’s sperm following percoll purification with Western analysis. Line 1: control wild type sperm protein, 20 ug; Line 2, 6: protein molecular weight ladder (bands of 20, 30, 40 and 50 kD); Line 3: homozygote SB-CAG-Venus sperm protein; Line 4: hemizygote SB-CAG-Venus sperm protein; Line 5: control wild type sperm protein, 10 ug. Venus protein molecular weight is approximately 30 kD.
(TIF)
